# Supplementary material for: Longitudinal investigation of changes in resting-state co-activation patterns and their predictive ability in the zQ175 DN mouse model of Huntington’s disease
Source: Sci Rep. 2023 Jun 23;13:10194. doi: 10.1038/s41598-023-36812-y (PMC10290061; doi:10.1038/s41598-023-36812-y)
Supplement: Supplementary file 1 — Supplementary Information. [file 41598_2023_36812_MOESM1_ESM.docx]

**Longitudinal investigation of changes in resting-state co-activation patterns and their predictive ability in the zQ175 DN mouse model of Huntington’s disease**

Mohit H. Adhikari^1,2,*^, Tamara Vasilkovska^1,2,*^, Roger Cachope^3^, Haiying Tang^3^, Longbin Liu^3^, Georgios A. Keliris^4^, Ignacio Munoz-Sanjuan^3^, Dorian Pustina^3^, Annemie Van der Linden^1,2^, and Marleen Verhoye^1,2^

^1^ Bio-Imaging Lab, University of Antwerp, Antwerp, Belgium

^2^ µNEURO Research Centre of Excellence, University of Antwerp, Antwerp, Belgium

^3^ CHDI Management/CHDI Foundation, Princeton, NJ, United States of America.

^4^ Institute of Computer Science, Foundation for Research & Technology - Hellas, Heraklion, Crete, Greece

*: These authors contributed equally to this work.

**Corresponding author:**

Mohit H. Adhikari

Bio-imaging Lab, University of Antwerp

Building UC1.16, Campus Drie Eiken,

Universiteitsplein, 1,

2610 Wilrijk, Antwerp, Belgium.

Email: mohit.adhikari@uantwerpen.be; Phone : +32 3265 9706

Keywords: Huntington’s disease, Animal models, Resting-state, functional MRI, Co-activation patterns, Classification, machine-learning


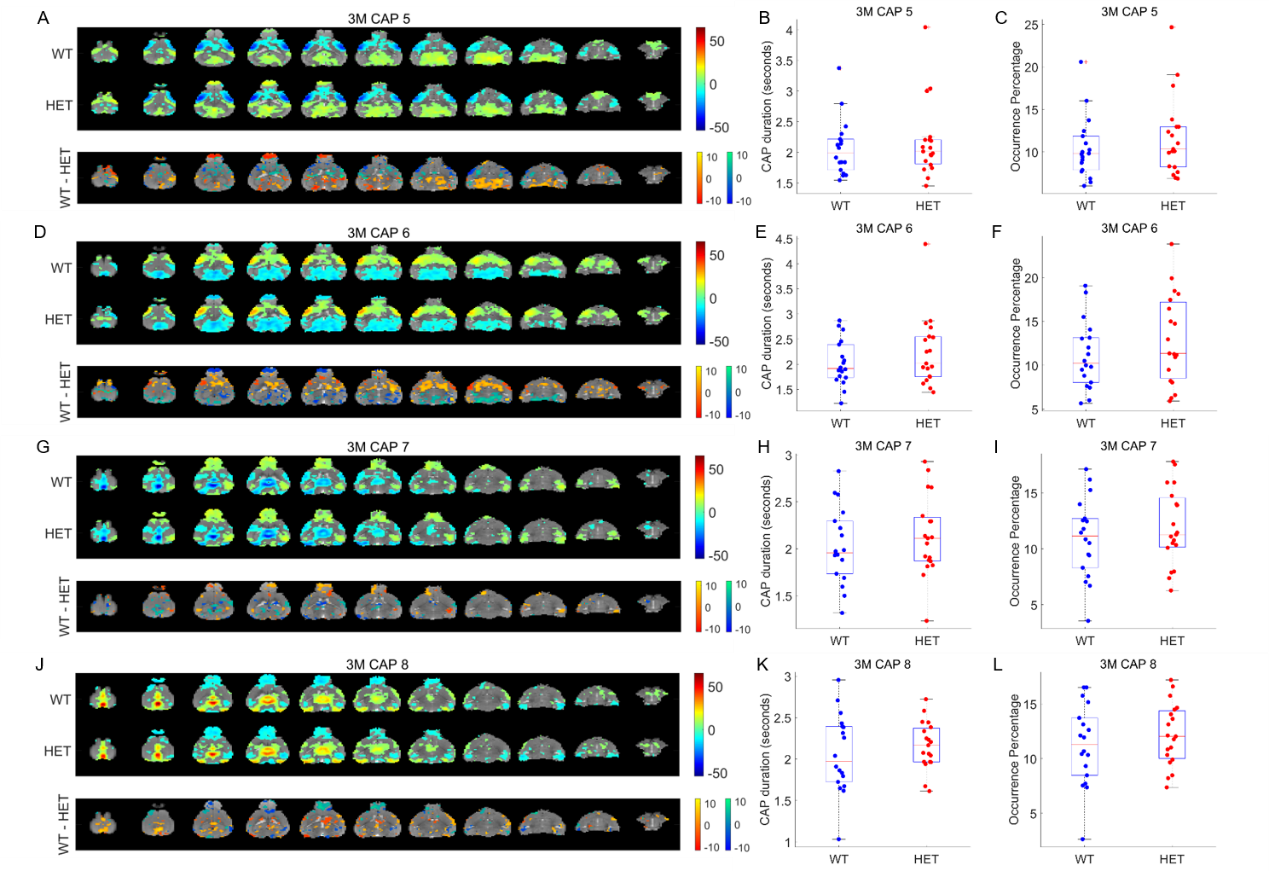


**Supplementary Figure 1:** Comparison of spatial and temporal properties of the other 4 out of 8 CAPs at the 3-month time point. **A, D, G, J:** top panels show the one-sample T-statistic maps of significantly (Bonferroni corrected, p<0.01) activated and deactivated voxels for each CAP obtained from its occurrences in the WT and HET portions of the combined image-series. Bottom panels show the 2-sample T-test statistic map of voxels with significant (FDR corrected, p<0.05) difference in the (de)activation between the WT and HET CAPs. We make these comparisons for all voxels that are significantly activated or deactivated in either the WT or the HET group. Red-yellow and blue-green colour bars refer to voxels that are co-activated and co-deactivated respectively in the WT group. Thus, positive (yellow, green) and negative (red, blue) T-statistic values respectively indicate significantly lower and higher magnitude of activation in the HET group compared to the WT group. **B, E, H, K:** Box-plots of comparisons of median duration, across subjects, of each CAP between WT & HET groups. **C, F, I, L:**  Box-plots of comparison of median occurrence percentage, across subjects, of each CAP between WT & HET groups. Black asterix indicates significant inter-group difference (p < 0.05, Wilcoxon rank-sum test, FDR corrected for 8 comparisons).


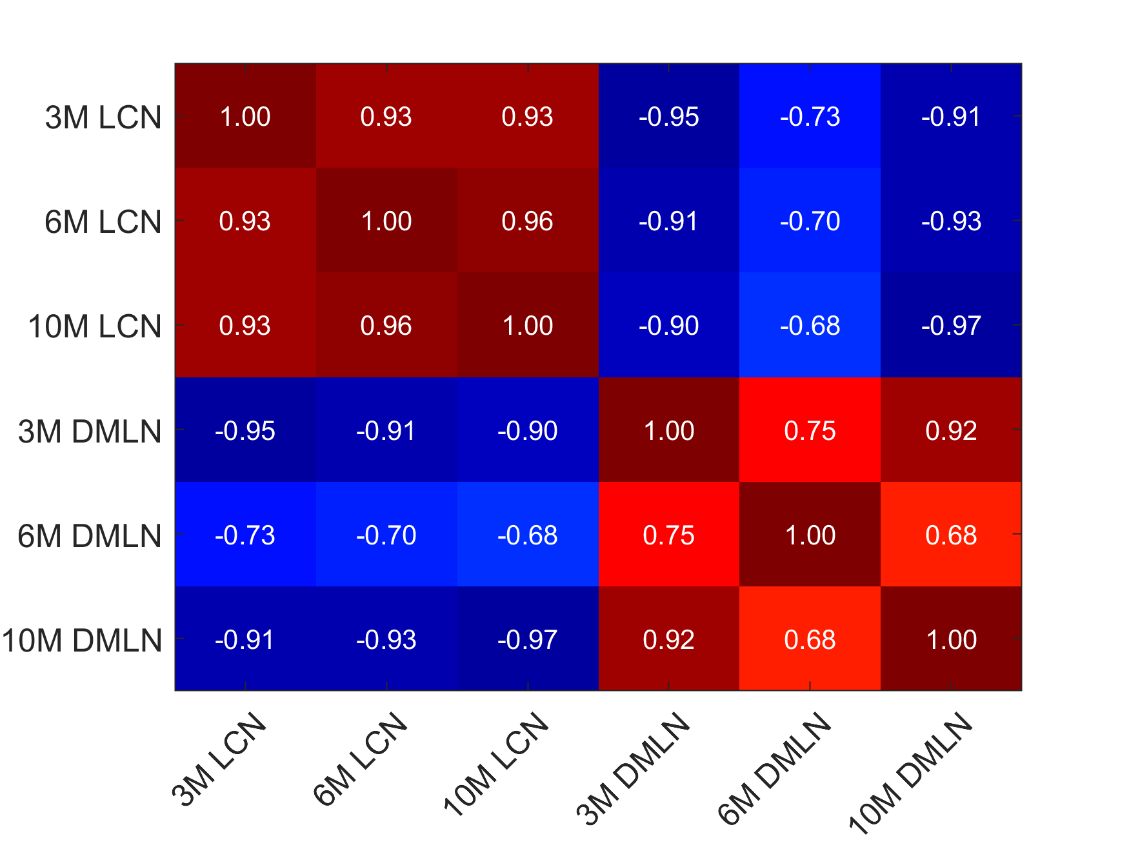


**Supplementary Figure 2:** Pearson’s correlation coefficient between spatial patterns of the LCN and DMLN CAPs identified at three time points.


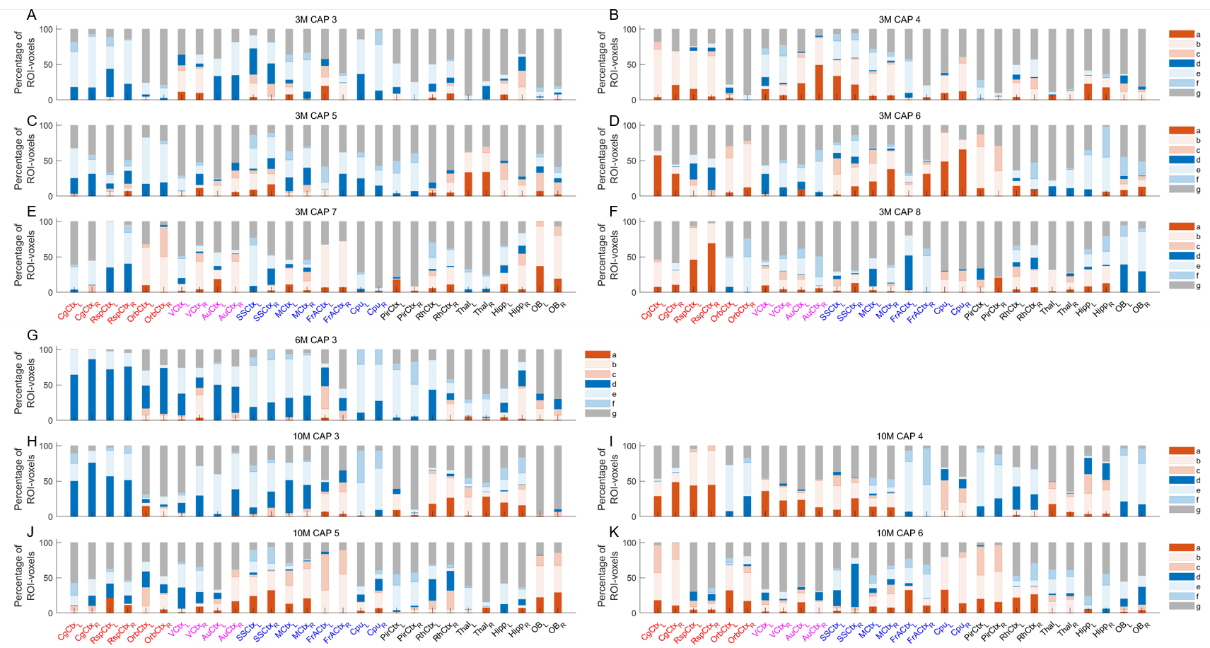


**Supplementary Figure 3:** Percentages of voxels per ROI belonging to one of 7 categories for the other-than-LCN-DMLN CAPs (A-F (3M), G (6M), H-K (10M)). The seven categories indicated by different colours identify voxels that show (a) significant co-activation and higher activation magnitude in WT, (b) significant co-activation and no significant inter-genotype difference in the activation magnitude, (c) significant co-activation and higher activation magnitude in HET, (d) significant co-deactivation and higher deactivation magnitude in WT, (e) significant co-deactivation and no significant inter-genotype difference in the activation magnitude, (f) significant co-deactivation and higher deactivation magnitude in HET, and (g) non-significant co-activation or co-deactivation during a CAP. Voxel-wise one-sample T-test (p < 0.01, Bonferroni corrected) and two-sample T-test (p < 0.05, FDR corrected) are performed across occurrences of a CAP within the concatenated genotypic image-series from all subjects to identify the voxels belonging to one of the 7 categories mentioned above and then divided among the 28 ROIs.


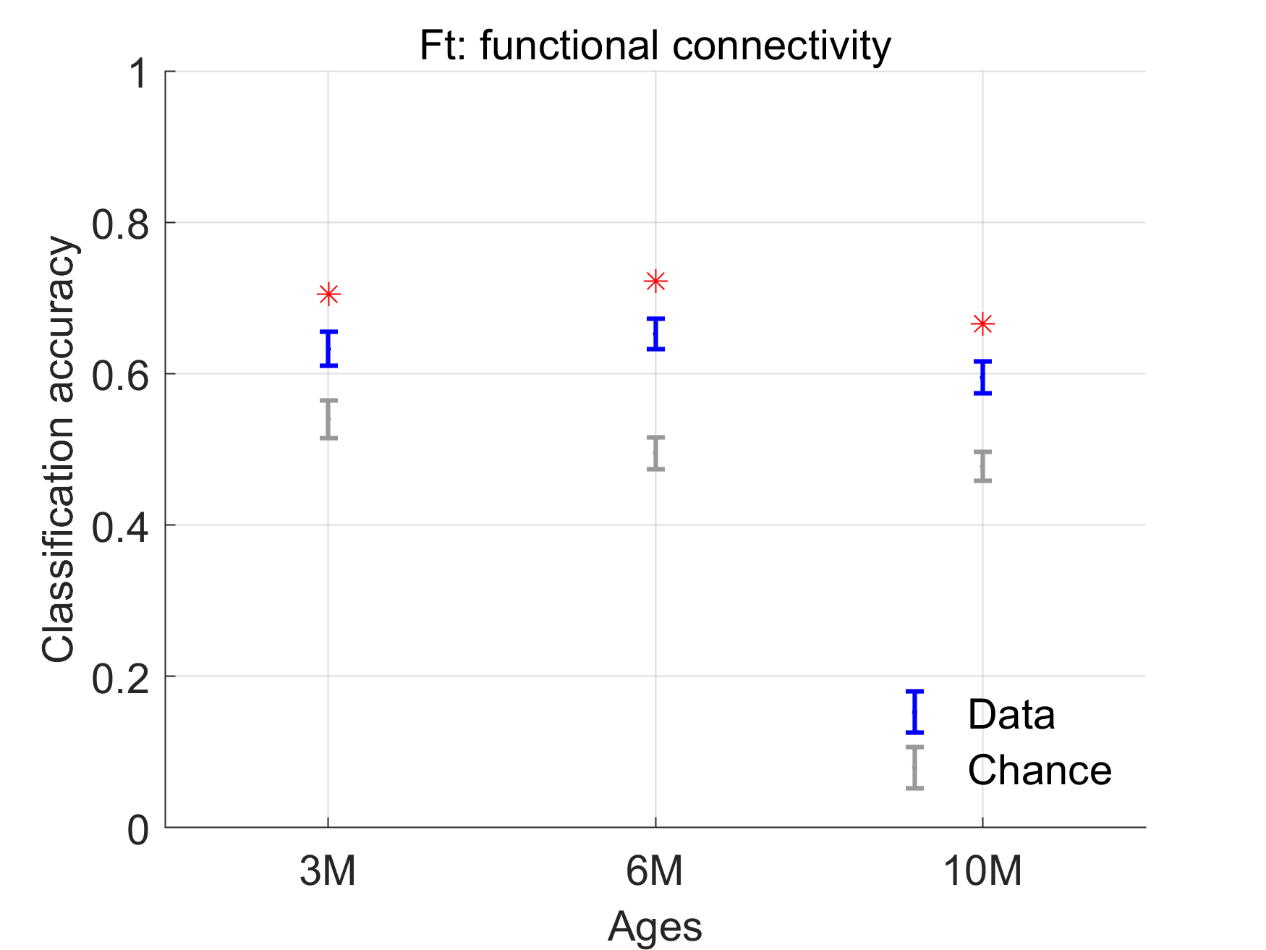


**Supplementary Figure 4:** Classification accuracy (blue, mean +/- SEM) using functional connectivity values between 26 regions of interest in both hemispheres at the 3-month, 6-month, and 10-month time points. The grey errorbars show the corresponding chance-level accuracy (mean +/- SEM) & red asterisk indicates significantly higher mean accuracy than the chance level, after correcting for all 3 comparisons (p < 0.05; FDR corrected).
